# Protective Macroautophagy Is Involved in Vitamin E Succinate Effects on Human Gastric Carcinoma Cell Line SGC-7901 by Inhibiting mTOR Axis Phosphorylation

**DOI:** 10.1371/journal.pone.0132829

**Published:** 2015-07-13

**Authors:** Liying Hou, Yuze Li, Huacui Song, Zhihong Zhang, Yanpei Sun, Xuguang Zhang, Kun Wu

**Affiliations:** 1 Department of Nutrition and Food Hygiene, School of Public Health, Harbin Medical University, Harbin, China; 2 Department of the Fourth Internal Medicine, The Fourth Hospital of Heilongjiang Province, Harbin, China; 3 Food Processing Institute, Heilongjiang Academy of Agricultural Sciences, Harbin, China; 4 Department of Internal Medicine, Hematology and Oncology, Harbin Children’s Hospital, Harbin, China; National Cheng Kung University, TAIWAN

## Abstract

Vitamin E succinate (VES), a potential cancer therapeutic agent, potently induces apoptosis and inhibits the growth of various cancer cells. Autophagy has been supposed to promote cancer cell survival or trigger cell death, depending on particular cancer types and tumor microenvironments. The role of autophagy in the growth suppressive effect of VES on gastric cancer cell is basically unknown. We aimed to determine whether and how autophagy affected the VES-induced inhibition of SGC-7901 human gastric carcinoma cell growth. SGC-7901 cells were treated with VES or pre-treated with autophagy inhibitor, chloroquine (CQ) and 3-methyladenine (3-MA). Electron microscopy, fluorescence microscopy and Western blot were used to study whether VES induced autophagy reaction in SGC-7901 cells. Western blot evaluated the activities of the mammalian target of rapamycin (mTOR) axis. Then we used 3-(4,5-dimethylthiazol-2-yl)-2,5-diphenyltetrazolium bromide (MTT) and flow cytometry to detect the level of cell viability and apoptosis. Collectively, our data indeed strongly support our hypothesis that VES treatment produced cytological variations that depict autophagy, increased the amount of intracellular green fluorescent protein—microtubule associated protein 1 light chain 3 (GFP-LC3) punctate fluorescence and the number of autophagic vacuoles. It altered the expression of endogenous autophagy marker LC3. VES activated the suppression of mTOR through inhibiting upstream regulators p38 MAPK and Akt. mTOR suppression consequently inhibited the activation of mTOR downstream targets p70S6K and 4E-BP-1. The activation of the upstream mTOR inhibitor AMPK had been up-regulated by VES. The results showed that pre-treatment SGC-7901 with autophagy inhibitors before VES treatment could increase the capacity of VES to reduce cell viability and to provoke apoptosis. In conclusion, VES-induced autophagy participates in SGC-7901 cell protection by inhibiting mTOR axis phosphorylation. Our findings not only strengthen our understanding of the roles of autophagy in cancer biology, but may also be useful for developing new treatments for gastric cancer patients.

## Introduction

Gastric carcinoma is among the most commonly diagnosed cancers in the world and is the second most frequent cause of cancer-associated mortality[1]. The incidence of gastric carcinoma and mortality from this disease have drastically decreased in most countries over the past 70 years, but gastric carcinoma is still the fourth most common cancer[2]. Gastric carcinoma is the third most common malignancy in China[3]. The major gastric carcinoma treatment modalities include surgery and chemotherapy, but survival among patients is low. The failure of chemotherapy is due to the development of drug resistance and toxicity. New strategies that overcome the abovementioned difficulties are required for treating gastric carcinoma.

Vitamin E succinate (VES; α-tocopheryl succinate) is a natural vitamin E (VE) derivative that shows potent anticancer effects on various cancers, including gastric carcinoma; VES is not toxic to normal tissues and cells in vitro and in vivo[4–10]. VES induces SGC-7901 human gastric carcinoma cell apoptosis by multiple signaling pathways, such as extrinsic Fas, mitogen-activated protein kinase (MAPK), and endoplasmic reticulum stress pathways[11–13].

Autophagy involves the degradation of dysfunctional and unnecessary cellular components and is related to various human diseases, especially cancer[14]. Autophagy, also known as macroautophagy, involves the transport of cytosolic components into the lysosomal lumen for degradation. Autophagy is important in preventing cellular damage and maintaining cellular homeostasis. Autophagy is involved in the suppression of human tumors[15–19]. Under metabolic stress, autophagy promotes cancer cell survival, but also triggers cell death[20, 21]. Thus, the effects of autophagy are contradictory; pathways involved in cell survival and death are promoted by autophagy[22]. Tumor cell lines treated with various chemotherapeutic drugs exhibit autophagy. Autophagy is upregulated in gastric cancer, as shown in previous studies[19, 23, 24]. Tumor cells are protected from the cytotoxic effects of cancer therapy by autophagy, which functions as the cell’s survival mechanism[25]. Autophagy serves an important function in stress response and cellular homeostasis maintenance and is regulated by a number of cross-talking signaling pathways[26]. Mammalian target of rapamycin (mTOR) is involved in autophagy and growth regulation; mTOR coordinates the balance regulation between cell development and autophagy under different cellular physiological conditions and environmental stress[27]. mTOR is a conserved serine/threonine kinase that is involved in the regulation of carcinogenic and metabolic events, such as autophagy, at certain key points[28]. mTOR stimulates protein synthesis by phosphorylating key translation regulators, such as ribosomal protein S6 kinase (p70S6K) and eukaryotic initiation factor 4E binding protein 1 (4E-BP-1)[29]. mTOR also prevents mammalian cell autophagy. Cell development suppression and autophagy are catabolic processes that are induced by mTOR inhibition[30]. Mammalian mTOR activation is controlled by the kinase cascade of the PI3K/ protein kinase B (Akt) signaling pathway[31] or by the reduction of phosphorylation of various protein kinases, such as p38 mitogen-activated protein kinase (p38 MAPK)[32].

We aimed to determine the occurrence of VES-induced autophagy in SGC-7901 cells and whether or not VES-induced autophagy could prevent cell death. The regulatory effects of the mTOR axis on autophagy in VES-treated cells were investigated.

## Materials and Methods

### Antibodies and reagents

VES (T3126), chloroquine (C6628), 3-methyladenine (M9281) and 3-(4,5-dimethylthiazol-2-yl)-2,5-diphenyltetrazolium bromide (MTT) (88417) were purchased from Sigma. Primary antibodies used for western blotting were rabbit anti-LC3 (Sigma, L7543), rabbit anti-Akt (Cell Signaling, 9272), rabbit anti-p-Akt (S473) (Cell Signaling, 9271S), rabbit anti-mTOR (Cell Signaling, 2972S), rabbit anti-p-mTOR (Ser2448) (Cell Signaling,5536S), rabbit anti-p70S6K (Cell Signaling,5707S), rabbit anti-p-p70S6K (T389) (Cell Signaling, 9205S), rabbit anti-4E-BP-1(Cell Signaling, 8594S), rabbit anti-p-4E-BP-1 (Thr37/46) (Cell Signaling, 9459S), rabbit antip38 MAPK (Cell Signaling,,8690S), rabbit anti-p-p38 MAPK (The180/Tyr1820) (Cell Signaling, 4092), rabbit anti-5'Adenosine monophosphate-activated protein kinase α (AMPKα) (Cell Signaling, 5831S), rabbit anti-p-AMPKα (Thr172) (Cell Signaling, 2535S) and rabbit anti-Actin (Santa Cruz Biotechnology, sc-1616). Alkaline phosphatase conjugated second antibody used for western blotting was anti-rabbit IgG (promega, S373B). Green fluorescent protein—microtubule associated protein 1 light chain 3 (GFP-LC3) plasmid was obtained from YRGene, China (Yrbio, VXY0542). All cell culture solutions were obtained from Thermo and all plastic-ware was obtained from Nunc, unless otherwise stated.

### Cell culture

Human SGC-7901 gastric carcinoma cells were obtained from Chinese academy of sciences cell resource center. Human SGC-7901 gastric carcinoma cells were cultured in a monolayer in Roswell Park Memorial Institute (RPMI)-1640 medium containing 10% fetal bovine serum (FBS), 1% penicillin (10,000 IU) and 1% streptomycin (10,000 μg/ml) in a humidified 5% CO_2_ incubator at 37°C. For experiments, the level of fetal bovine serum was reduced to 2% in order to maintain the cell survival and inhibit the cell proliferation to the maximum extent. VES was dissolved in sterile absolute ethanol to produce a 10 mg/mL stock solution, which was subsequently diluted in RPMI-1640 complete condition media to yield different concentrations. An equal amount of ethanol was used as solvent control.

### Cell viability assay

Cell viability was measured by MTT assay. Cells were plated at 8,000 cells per well in 96-well plates. The MTT solution was dissolved in a culture medium to yield a final concentration of 5 mg/mL and added to each well at the end of VES treatment. The plates were incubated for 4 h. Dimethyl sulfoxide (Beyotime, China) was added to the plates to solubilize the MTT tetrazolium crystals. The optical density was determined at 570 nm using a Benchmark Plus microplate reader (Bio-Rad, USA).

### Cell cycle analysis

Cells were fixed using ice-cold 75% ethanol in phosphate buffered saline (PBS) and were incubated with 50 μg/mL propidium iodide (PI), 3.8 mmol/L sodium citrate, and 0.5 μg/mL RNase A (The CycleTEST PLUS DNA Reagent Kit, BD, USA) at 4°C for 3 h. The sample was then analyzed by flow cytometry for 488 nm excitation and 590~630 nm emission (Becton Dickinson, USA). Cell cycle phase was analyzed with the CellQuest software.

### Transmission electron microscopic analysis

The SGC-7901 cells were harvested by trypsinization (Beyotime, China) after 24 h of treatment, washed twice with PBS, fixed with 2.5% glutaraldehyde (Beyotime, China) in 0.1 mol/L PBS (pH 7.4) at room temperature for 90 min, and post-fixed in 1% osmium tetraoxide for 30 min. The cells were washed with PBS, progressively dehydrated in a 10% graded series of 50% to 100% ethanol and propylene oxide, and embedded in Epon 812 resin. The blocks were cut into ultrathin sections using a microtome. The sections were then stained with saturated uranyl acetate and lead citrate. The cell ultrastructure was observed by transmission electron microscopic (H-7650, HITACHI, Japan).

### Autophagy analysis by GFP-cytosolic microtubule associated with protein LC3 distribution

The SGC-7901 cells were transiently transfected using the Lipofectamine 2000 reagent (Invitrogen, 11668–027), according to the manufacturer's recommendations. The cells were transfected with a control vehicle or a GFP–LC3 fusion protein expression vector (pEGFP-C1-LC3). The transfected cells were harvested after VES treatment, and LC3 localization and autophagosome formation were analyzed by fluorescence microscopy (Nikon, Japan).

### Western blot analyses

The cells were lysed in the lysis buffer (150 mM NaCl, 0.1% NP-40, 0.5% sodium deoxycholate, 0.1% SDS, 50 mM-Tris, 1mM-dithiothreitol, 5 mM-Na_3_VO_4_, 1mM-phenylmethylsulfonyl fluoride, 10 μg/ml trypsin, 10 μg/ml aprotinin, 5μg/ml leupeptin; pH 7.4). After incubation for 30 min at 4°C, the sample was centrifuged at 15,000 *g* for 8 min at 4°C, and the supernatant was collected as whole cell lysate and stored at -80°C until use. Equivalent amounts of proteins were separated on 10% SDS–PAGE gels and transferred to a nitrocellulose membrane. Western blot analyses were performed using LC3-II, AMPK, p-AMPK, p38 MAPK, p-p38 MAPK, Akt, p-Akt, mTOR, p-mTOR, p70S6K, p-p70S6K, 4E-BP-1, p-4E-BP-1, and β-actin antibodies. The membrane was incubated with the secondary alkaline phosphatase-conjugated IgG and detected using the Western Blue Stabilized Substrate for alkaline phosphatase (Promega, USA). The bands were analyzed using ChemiImager 4000 (Alpha Innotech, USA).

### Apoptosis assessment

The combined effects of VES and autophagy inhibitors on the cell nuclear morphology were analyzed by Hoechst 33342 (Beyotime, C1022) staining. SGC-7901 cells (1 × 10^5^ cells/well; 24 wells) were pretreated with autophagy inhibitors chloroquine (CQ) and 3-methyladenine (3-MA). Cells were incubated with VES (20 μg/mL) in growth medium at 37°C for 24 h. The cells were stained with Hoechst 33342 (1 μg/mL) for 15 min after incubation and washed twice with PBS. The cells were observed under a confocal microscope (Nikon, Japan).

Apoptotic cell death was observed using Annexin V-FITC Apoptosis Detection kit (BD, USA) according to the manufacturer's recommendation. The cells were washed twice with cold PBS after the treatment and collected by centrifugation. The pellet was resuspended in 1× binding buffer and stained with 5 μL fluorescein isothiocyanate-labeled Annexin V at 4°C for 15 min in the dark. PI (10 μL) was added at 4°C for 5 min in the dark. Cells were analyzed by flow cytometry (Becton Dickinson, USA) with the CellQuest software. The levels of apoptosis were determined using fluorescence detector. Fluorescence of FITC was detected at Ex = 488 nm and Em = 525 nm. Fluorescence of PI was detected at Ex = 488 nm and Em = 615 nm.

### Statistical analyses

Statistical analyses were conducted by one-way analysis of variance. The data are presented as mean ± standard error. Experiments were performed at least three times. Results were considered statistically significant at *p* < 0.05.

## Results

### VES inhibited gastric carcinoma cell SGC-7901 viability

**Fig 1:**
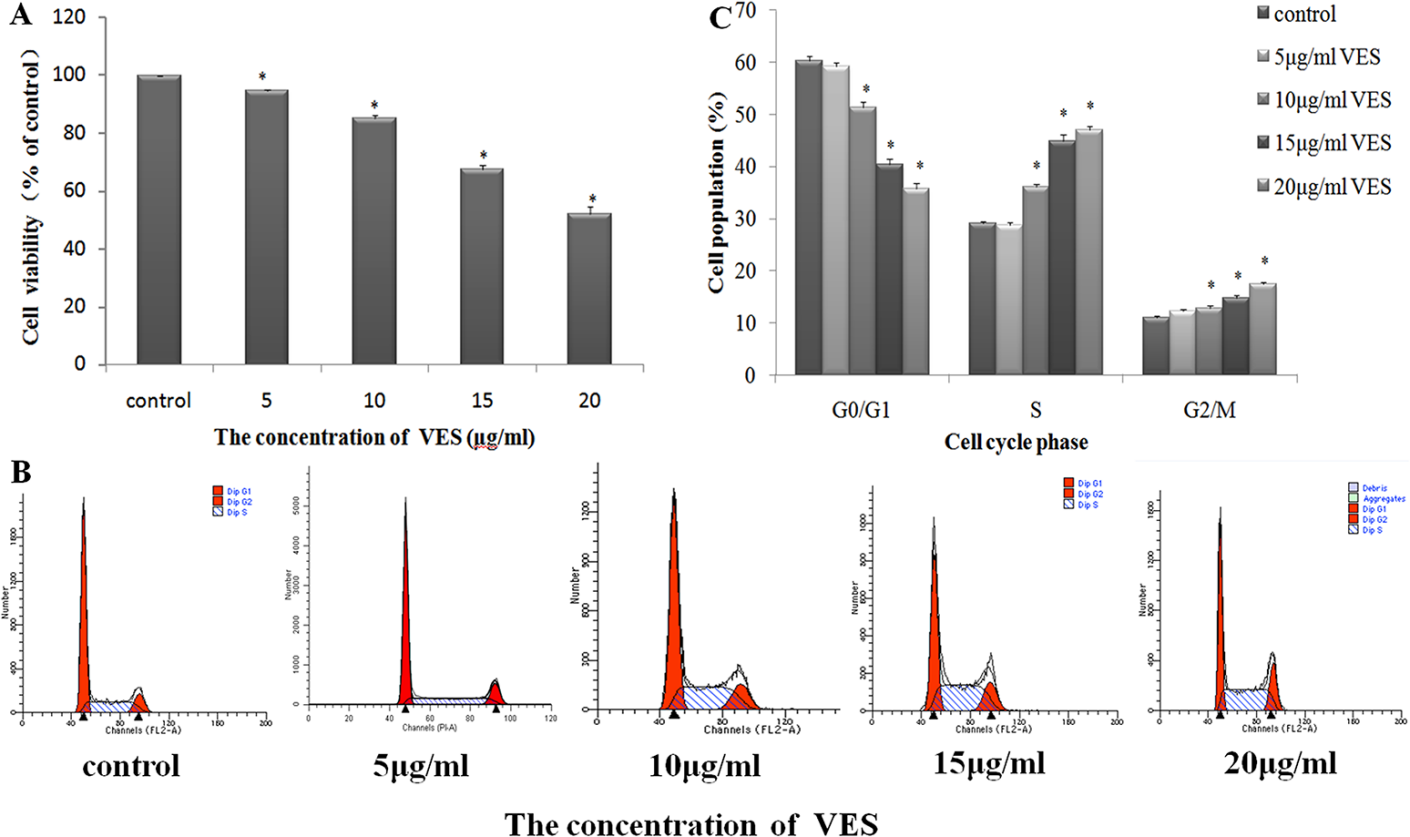
Inhibition of gastric cancer cell SGC-7901 viability by VES. (A) Incubation of VES for 24 h decreased cell viability in a concentration-dependent manner as determined by MTT assay in SGC-7901. (B) Cells were treated with or without different concentrations of VES for 24 h. DNA contents were determined by flow cytometry analysis. (C) DNA histogram shows the accumulation of S and G2/M phases cells induced by VES. *p < 0.05 means significantly different from the control group.

The changes in 3-(4,5-dimethylthiazol-2-yl)-2,5-diphenyltetrazolium bromide (MTT) tetrazolium salt formation in VES-treated SGC-7901 cells were examined to study the effect of VES on gastric carcinoma cell viability. VES significantly reduced MTT tetrazolium salt formation in cells in a dose-dependent manner (Fig 1A). SGC-7901 cell viability was inhibited by approximately 5.3%, 14.7%, 34.4%, and 47.8% compared with the vehicle control (0.01% v/v Anhydrous ethanol) under 5, 10, 15, and 20 μg/mL VES treatments, respectively, for 24 h. Flow cytometry-based cell cycle analysis was performed to analyze the anti-mitogenic activity under VES treatment. VES at 20 μg/mL increased the number of SGC-7901 cells arrested at the S and G2/M phases, and this effect was dose dependent. (Fig 1B and 1C).

### VES induced an autophagy in SGC-7901 cells

**Fig 2:**
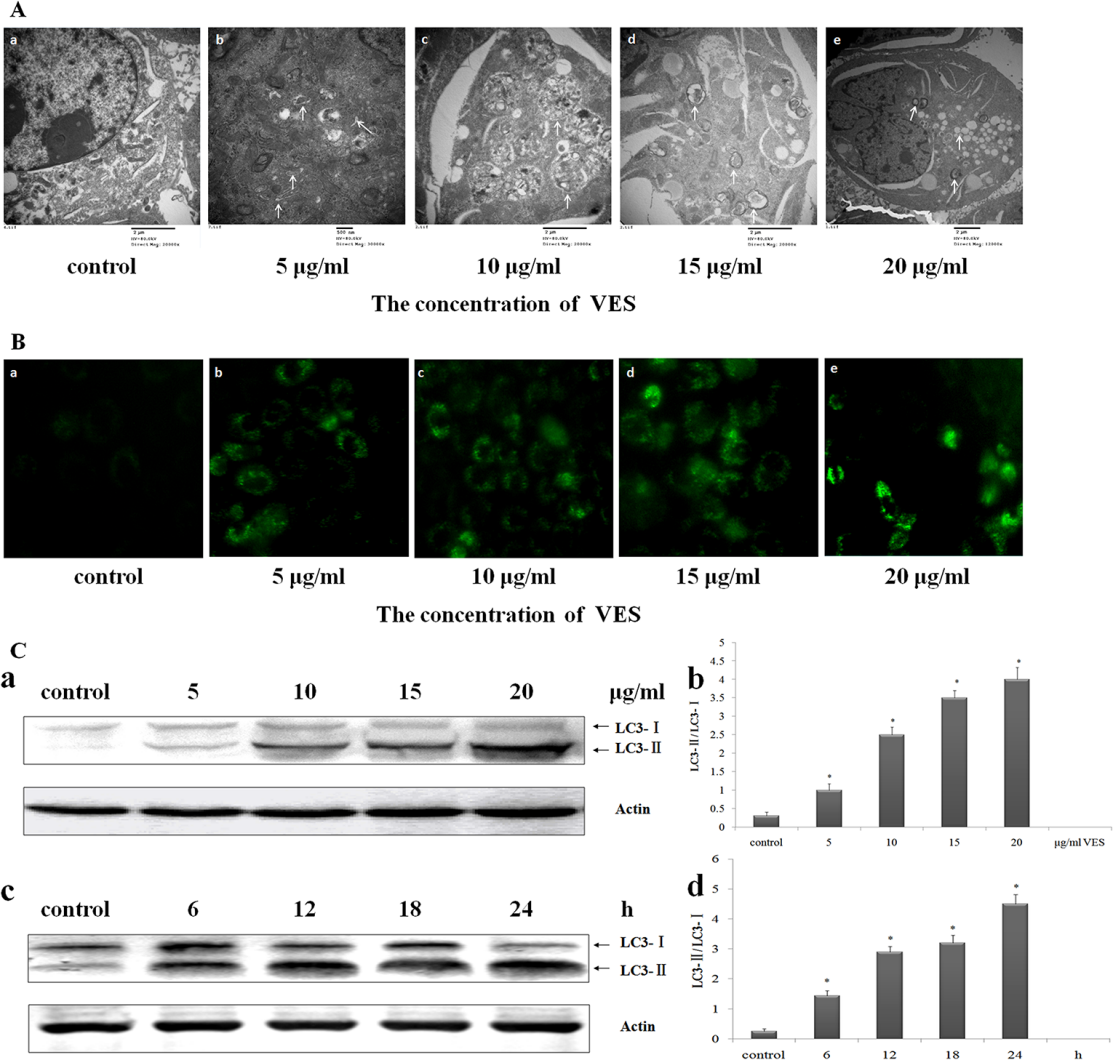
VES induces an autophagic response in SGC-7901 cells. (A) Ultrastructural observations in SGC-7901 cells with transmission electron microscope. (a) Untreated SGC-7901 cells exhibited normal of cytoplasm, cell organelles, and nuclei morphologies. VES-treated SGC-7901 cells showed characteristic ultrastructural morphology of autophagy, (b to c) a significant number of autophagic vacuoles, (d) a giant autophagic vacuole filled with abundant degraded organelles, (e) autophagic vacuoles fused with multivesicular endosome. (a, c, & d, 20 000×; b, 30 000×; e, 12 000×). Typical changes were indicated by arrows. (B) Treatment of SGC-7901 cells with VES for 24 h prominently enhanced formation of autophagic vacuoles and fluorescent intensity, as determined by transfection of GFP-LC3 (C) Protein levels of LC3-I and LC3-II were measured using Western blot analysis. Data was representative of three individual experiments with similar results. (a) SGC-7901 cells were treated with 0, 5, 10, 15, and 20 μg/mL VES for 24 h. (c) SGC-7901 cells were treated with 20 μg/mL VES for 0, 6, 12, 18, and 24 h. (b) and (d) Relative densitometry of protein expression was determined by LC3-II protein densitometry with LC3-I. Actin was used as a loading control.* means *p* < 0.05 compared with control.

Whether or not VES induced autophagy in SGC-7901 cells was investigated. Autophagic bodies or autophagosomes comprising double- or single-membrane structures are located in the cytoplasm. Such autophagosomes were analyzed for topical autophagic changes by electron microscopy. The electron micrographs of normal SGC-7901 cells revealed intact cells, distinct nuclei and nuclear membranes, rough endoplasmic reticula, and homogeneous cytoplasms (Fig 2Aa). In VES-treated SGC-7901 cells, membrane-bound autophagosomes were observed in the cytoplasm (Fig 2Ab to 2Ae). Electron micrographs revealed that VES at 5 and 10 μg/mL increased the number of autophagosomes containing mitochondria, endoplasmic reticulum (ER) membranes, and ribosomes in SGC-7901 cells (Fig 2Ab to 2Ac). Partial degradation of the contents of late-degradation autophagic vacuole was evident, and the engulfed rough ER was degraded in cells treated with 10 μg/mL VES (indicated by arrows). The contents of numerous small vesicles (indicated by arrows) showed the fusion between autophagic vacuole and the multivesicular endosome (Fig 1Ad and 1Ae). VES-treated cells showed more autophagosomes than untreated cells (Fig 2Aa).

Light chain 3 (LC3) localization in cells transfected with green fluorescent protein (GFP)-labeled LC3 (GFP–LC3) was determined. Cytoplasm of transfected cells showed the distribution of GFP–LC3 dots (Fig 2B). The distribution of green dots in the control group was dispersed and indistinct. The punctate fluorescence in the VES treatment groups increasingly coagulated and intensified with increasing VES concentration. The increase in number and fluorescence intensities of the GFP-LC3 dots or vacuoles was induced by VES after 24 h of treatment (Fig 2B).

LC3 protein is an autophagy marker and its expression was analyzed to determine how VES affected autophagy in the cells. The LC3-II protein levels significantly increased with increasing VES concentration in a time-dependent manner (Fig 2C). The processed form of LC3-II also increased, thereby indicating an increase in autophagy incidence. Hence, VES could potentially induce autophagy in SGC-7901.

### EFfect of VES on mTOR-related signaling pathways

**Fig 3:**
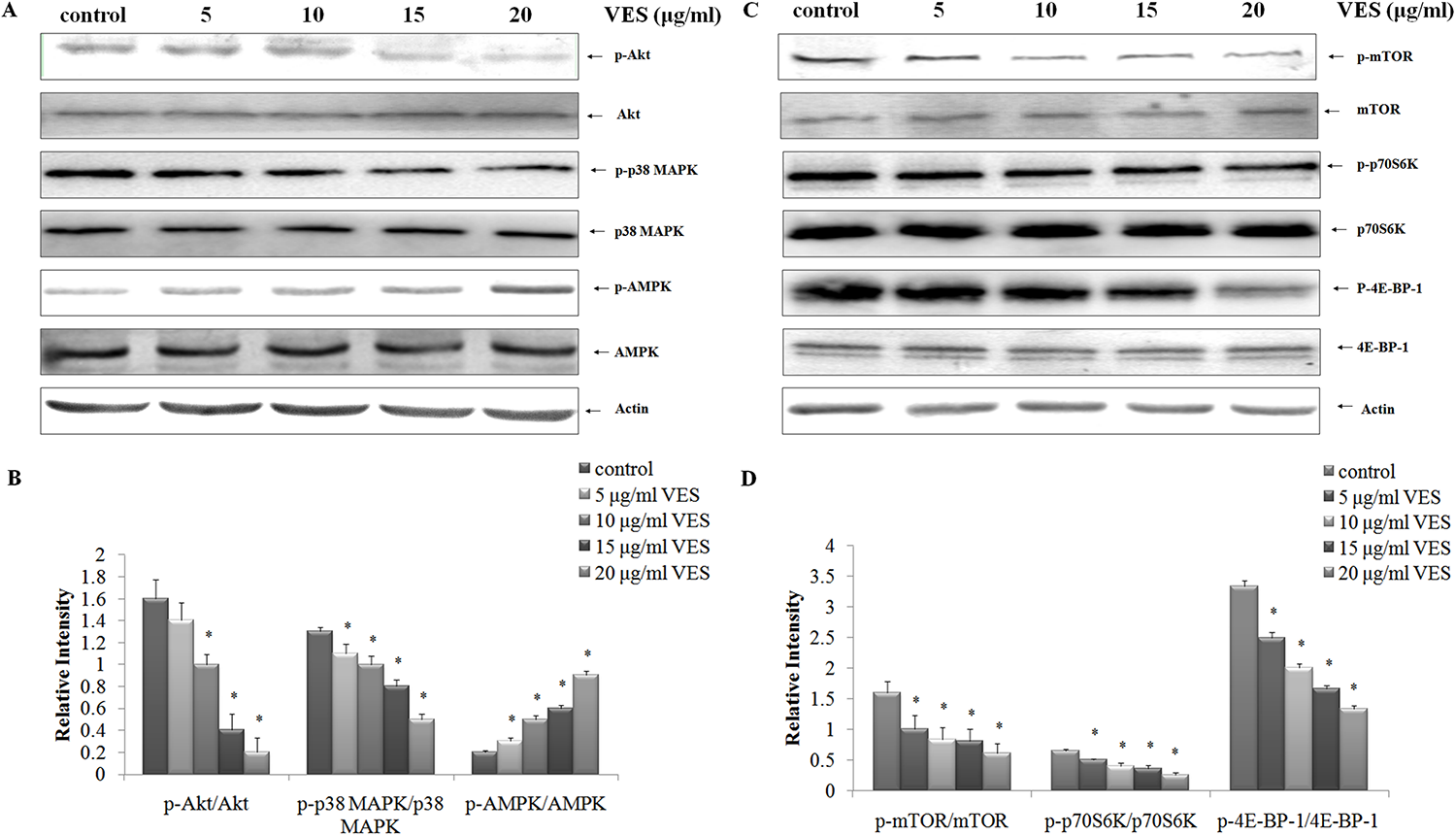
Effects of VES on mTOR axis. SGC-7901 cells were treated with 0, 5, 10, 15, and 20 μg/mL VES for 24 h, and activation of p38 MAPK (p-p38 MAPK), Akt (p-Akt), AMPK (p-AMPK), mTOR (p-mTOR), p70S6K (p- p70S6K), and 4E-BP-1 (p-4E-BP-1) were examined using Western blot. The data in (A) and (C) were representative of three individual experiments with similar results. The data in (B) and (D) were expressed as mean ± S.D from three individual experiments. Actin was used as a loading control. * means *p* < 0.05 compared with control.

mTOR is an important component of the autophagy-related pathway. mTOR activation was analyzed to determine the molecular mechanism underlying VES-mediated autophagy induction in SGC-7901 cells. VES effects on mTOR and p-mTOR (Ser2448) expressions were analyzed. In particular, VES effects on phosphorylated p70S6 kinase at Thr-389 and the 4E-BP-1 at Thr37/46 were determined. mTOR, p70S6K, and 4E-BP-1 phosphorylation levels decreased with increasing VES concentration (Fig 3C and 3D).

Western blot analysis results showed that VES inhibited the phosphorylation of mTOR and its downstream targets, p70S6K and 4E-BP-1. Such inhibition triggered autophagy progression. We also investigated how VES affected the activation of the upstream signals of mTOR inhibitor, AMP-activated protein kinase (AMPK), and mTOR activators, namely, Akt and p38 MAPK. The levels of Akt, p38 MAPK, and AMPK in VES-treated SGC-7901 cells were analyzed. Phosphorylations of Akt (p-Akt) and phosphorylation of p38 MAPK (p-p38 MAPK) decreased with increasing VES concentrations. In addition, activated p-AMPK levels were up-regulated in VES-treated cells compared with control group (Fig 3A and 3B). However, VES failed to affect total steady state protein levels.

### Blocking early and late autophagy stages enhanced the antiproliferative effect of VES

The function of autophagy in VES-treated SGC-7901 cells was investigated by inhibiting autophagy using 3-methyladenine (3-MA) and chloroquine (CQ). VES-treated or untreated SGC-7901 cells were exposed to 10 mM 3-MA or 20 μM CQ. The changes in cell morphology and viability and cell cycle variations were analyzed.

Untreated control cells adhered well and displayed normal SGC-7901 cell morphology. When autophagy was inhibited, the number of dead cells increased and the cells became round and detached (S1 Fig).

**Fig 4:**
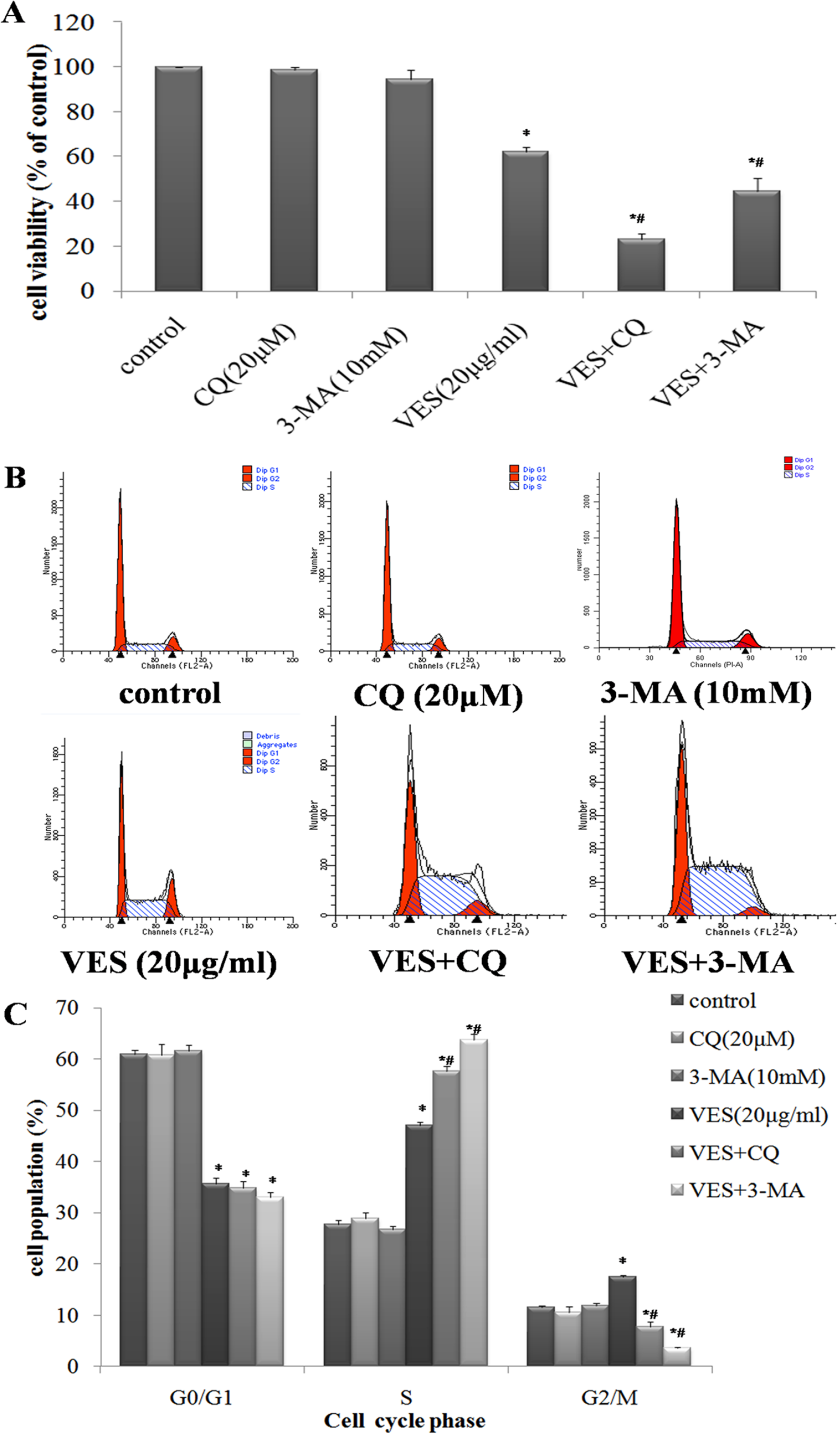
Autophagy inhibition enhances VES-induced viability suppression in SGC-7901. SGC-7901 cells were exposed to VES, CQ, or 3-MA alone or in presence of 10 mM 3-MA or 20 μM CQ for 24 h. (A) MTT assay show that combined action of both VES and CQ and VES and 3-MA significantly suppress cell viability compared with VES treatment alone (*, compared with control group, *p* < 0.05; #, compared with VES group, *p* < 0.05). (B) and (C) DNA histogram shows that accumulation of S phase cells induced by VES was enhanced by CQ and 3-MA (*, compared with control group, *p* < 0.05; #, compared with VES group, *p* < 0.05).

MTT assay and cell cycle analysis were performed to determine the function of autophagy in VES-induced inhibition of SGC-7901 cell viability. The SGC-7901 cells were subjected to the abovementioned methods. Results of the MTT assay showed that the combination of VES with either CQ or 3-MA led to a significantly higher suppression of cell viability than VES alone (*p* < 0.05, Fig 4A).

The early stage of cell autophagy was firstly blocked by 3-MA, and then the cell cycle was analyzed by flow cytometry. The group treated with VES and 3-MA showed a significantly higher number of cells arrested at S phase than both the control group and VES alone (*p* < 0.05, Fig 4B and 4C). CQ blocked late-stage autophagy and increased the cytostatic effect of VES. The group treated with VES and CQ showed a significantly higher number of cells arrested at S phase than both the control group and VES alone (*p* < 0.05, Fig 4B and 4C).

### The role of autophagy on VES-induced apoptosis

The biological activity of VES-induced autophagy in apoptosis was determined. The possible enhancement of VES-induced apoptosis by autophagy was determined using a confocal microscope and by flow cytometry after staining the cells with either Hoechst 33343 or Annexin V-FITC/PI.

**Fig 5:**
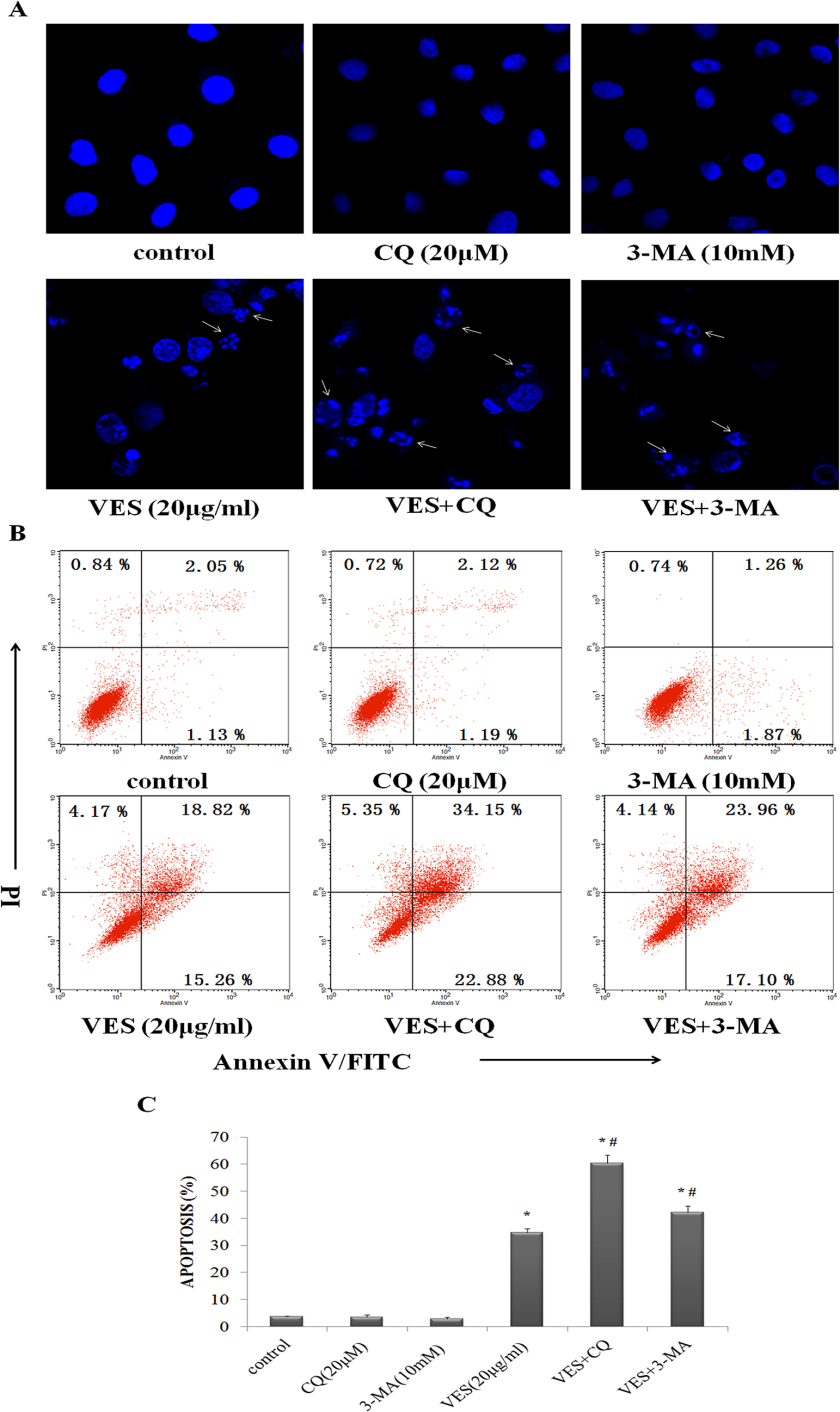
Autophagy inhibition enhances VES-induced apoptosis in SGC-7901. (A) SGC-7901 cells were exposed to VES, CQ, or 3-MA alone or in presence of 10 mM 3-MA or 20 μM CQ for 24 h, respectively, stained with Hoechst 33343, and visualized under a confocal microscopy (1000×) (B) SGC-7901 human gastric cancer cells were treated as (A), stained with Annexin V-FITC/PI, and detected through flow cytometry analysis. The significance of the obtained four quadrants: FITC Annexin V-negative/PI-negative cells as viable cells, FITC Annexin V-positive/PI-negative cells as early apoptotic cells, FITC Annexin V-positive/PI-positive cells as late apoptotic cells, and FITC Annexin V-negative/PI-positive cells as necrosis cells. (C) Columns: mean of triplicate treatments; bars: ± SD. (*, compared with control group, *p* < 0.05; #, compared with VES group, *p* < 0.05).

The effects of the combined treatment of autophagy inhibitors and VES on cellular apoptosis in SGC-7901 cells were analyzed. A higher number of cells in the group treated with a combination of CQ, 3-MA, and VES displayed apoptosis-related morphological changes, such as cell shrinking and chromatin condensation and crescent formation / margination compared with the untreated group or the group treated with CQ or 3-MA alone. DNA fragmentation and apoptotic body formation were observed in the VES + CQ and VES + 3-MA groups (Fig 5A). Flow cytometric analysis results also showed that treatment with CQ (20 μM), 3-MA (10 mM), or VES (20 μg/mL) induced apoptosis in 4.31%, 3.53%, or 34.08% of the cells, respectively. Combination of CQ, 3-MA, and VES at the abovementioned concentrations induced apoptosis in 57.03% or 41.06% of the cells (Fig 5B and 5C). The combination of CQ and VES or 3-MA and VES showed higher cooperative effect on the occurrence of VES-induced apoptosis than each agent applied alone. These data confirmed that VES-induced autophagy contributed to the prevention of VES-induced apoptosis in human gastric carcinoma cells.

## Discussion

We focused on VES in this study. A VE analog can substantially enhance apoptosis in malignant cells, as shown in vitro and in vivo studies using various species and tissues[13, 33–37].

SGC-7901 cell viability was inhibited under VES treatment in a dose-dependent manner (Fig 1A). Our results are in agreement with previously reported findings[10, 13]. Flow cytometry cell cycle analysis showed that the number of cells arrested in the S and G2/M phases increased under VES treatment (Fig 1B and 1C), thereby suggesting that inhibition of cell cycle progression may a mechanism underlying the antiproliferative effect of VES.

According to a previously report researched by Karim MR[38], vitamin E act as a novel enhancer of macroautophagy. VES, as a VE analog, has been proved as a autophagy inducer in human gastric cancer by our study. Transmission electron microscopy (TEM) is a conventional method for observing autophagy; the phenomenon of autophagy was initially discovered using this method[39]. Autophagy is also detected by observing autolysosome formation under GFP–LC3 transfection[39, 40]. The microtubule-associated protein 1 (MAP1)–LC3, which is important in autophagy, is the first mammalian protein to be detected in autophagosome membranes[24, 40, 41]. A copious amount of LC3B is synthesized during autophagy. Thus, LC3-II is an important autophagy marker.[42]. The abovementioned strategies were used to demonstrate the induction effect of VES on SGC-7901 cell autophagy (Fig 2).

mTOR is a major component of autophagy regulation and integrates numerous extracellular signals, including glucose, amino acids, growth factors, and energy status[43]. The important function of mTOR as an autophagy repressor suggests that mTOR activity downregulation efficiently triggers autophagic response[43–45]. mTOR phosphorylation increases the levels of downstream targets, such as p70S6K and 4E-BP-1 to regulate many different cellular processes[46, 47]. The p70S6K phosphorylation level is important in the initiation of the translation of proteins associated with cell growth and proliferation. The p70S6K, 4E-BP-1, and mTOR phosphorylations decreased in VES-treated SGC-7901 cells, suggesting that VES inhibited mTOR activity (Fig 3C and 3D).

The serine/threonine protein kinase Akt (protein kinase B) mediates mTOR activity[48], and positively regulates mTOR. VES-induced inhibition of Akt phosphorylation was detected in a dose-dependent manner in our studies. The mTOR pathway is regulated by the MAPK pathway, as discussed in a previous study[49]. We monitored p38 MAPK phosphorylation in the MAPK signaling pathway in VES-treated SGC-7901 cells. VES markedly suppressed p38 MAPK phosphorylations, and such inhibitory effect results in autophagy. AMPK activation decreased mTOR activity[50], thereby indicating that AMPK is a negative mTOR regulator. AMPK phosphorylation was up-regulated by VES treatment (Fig 3A and 3B).

Inhibition of mTOR phosphorylation and its signal transduction by VES may partly promote potent autophagy-inducing activity. The results implied that VES induced autophagy via the mTOR axis.

Autophagic cell death is an alternative cell death pathway for the chemotherapy of different cancer cells, which often develop resistance against drugs that activate the apoptotic pathway[51]. Previous studies have confirmed the dual function of autophagy in cell death. Autophagy promotes cell survival, but also induces cell death by promoting the extensive digestion of intracellular organelles[52, 53]. The function of autophagy in VES-treated SGC-7901 cells was investigated. Autophagy was inhibited by CQ and 3-MA. CQ is an antimalarial drug that inhibits autophagy at the late stage by blocking the fusion between autophagosome and lysosome fusion and by enzymatic autophagosomal cargo digestion[54]. Higher CQ concentrations could reduce the viability of tumor cells[55, 56], but the CQ concentration used in this study failed to induce tumor cell death in vitro (Figs 4 and 5). 3-MA is a PI3K inhibitor that inhibits autophagy at the early stage and is a common inhibitor of autophagy[57]. 3-MA effectively inhibited autophagy at the early stages[58] and also failed to induce tumor cell death (Figs 4 and 5). Autophagy blockage by either CQ or 3-MA enhances SGC-7901 cell apoptosis (Fig 5) and suppresses cell viability (Fig 4), respectively, under in vitro application of VES. The augmentation of tumor cell death by CQ and 3-MA treatments were correlated with the abilities of these compounds to block the process of autophagy. Thus, autophagy inhibition is a mechanism underlying the major antineoplastic effects of CQ and 3-MA at the abovementioned concentrations. These results suggested that VES-induced autophagy is used as a means for adaptation in SGC-7901 cells. Combination of VES and autophagy inhibitors increases the effects of therapeutic methods on the induction of tumor cell apoptosis.

**Fig 6:**
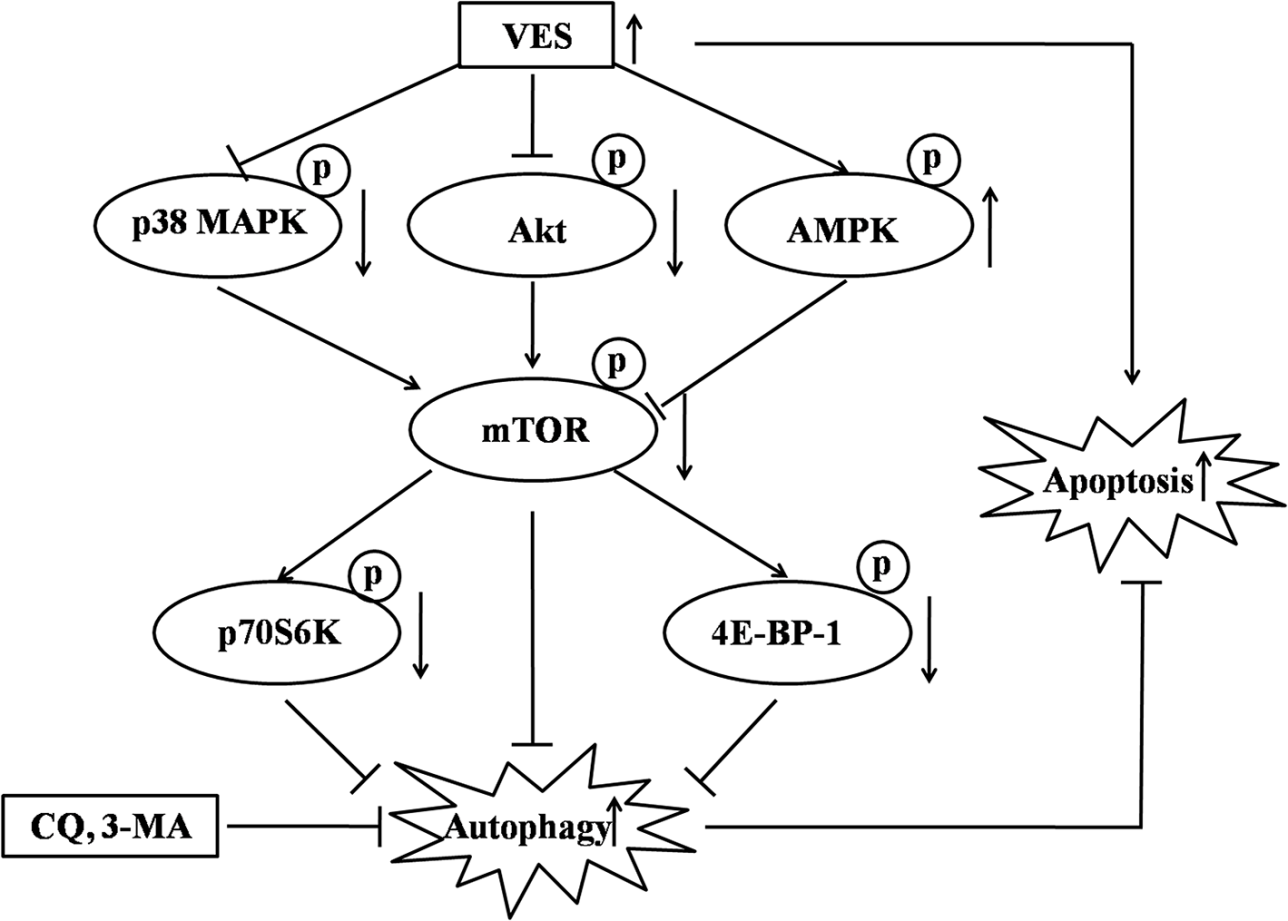
Hypothetical model of VES autophagy mechanism in human gastric carcinoma SGC-7901 cells. VES induced the suppression of both Akt and p38 MAPK, and up-regulated AMPK, leading to the inhibition of mTOR activity. And downstream regulators p70S6K and 4E-BP-1 were suppressed consequently, resulting in autophagy in SGC-7901 cells. VES cooperated with autophagy inhibitors to induce increased anticancer effect compared with VES alone.

VES induces autophagy by inhibiting the mTOR axis pathway in human gastric carcinoma SGC-7901 cells as depicted in Fig 6. VES induced the suppression of both Akt and p38 MAPK, and up-regulated AMPK, leading to the inhibition of mTOR activity. And downstream regulators p70S6K and 4E-BP-1 were suppressed consequently, resulting in autophagy in SGC-7901 cells. VES cooperated with autophagy inhibitors to induce increased anticancer effect compared with VES alone. Identifying the mTOR signaling transduction pathway clarified the molecular mechanisms that cause autophagy-mediated cell viability inhibition by antitumor agents and may contribute to the creation of novel therapeutic strategies for tumor growth suppression. Information is lacking on the detailed mechanisms that mediate the activation of kinases related to mTOR. We present an important perspective on cancer cell response to VES.

This study has several limitations. Autophagy was inhibited only using pharmacological approaches, which blocked the process of autophagy at early and late stages. Genetic investigations should be conducted to further verify the results of this study. The siRNA specific to the autophagy-related gene 7 (ATG7) must be applied to inhibit ATG7 expression and block autophagy at the initial stages.

We showed for the first time that VES induce macroautophagy. Such autophagy in gastric carcinoma cells counteracted the antiproliferative effects of VES. The autophagy induction in VES-treated SGC-7901 cells was mediated by the down-regulation of mTOR pathway. The use of an autophagy inhibitor may enhance VES anti-cancer effects. We presented new perspectives on the mechanism underlying VES-induced cell death and discussed the potential use of combined VES and autophagy inhibitors in cancer chemotherapy.

## Supporting Information

S1 Fig Morphological observation under a phase contrast microscope The SGC-7901 cells were seeded in six-well flat bottom plates. VES-treated or untreated SGC-7901 cells were exposed to 10 mM 3-MA or 20 μM CQ. The morphologies of the SGC-7901 cells were observed under a phase contrast microscope (Nikon, Japan) after 24 h of treatment. Bars: 100 μm.
(TIF)
