# Supplementary figures and images for: Protective Macroautophagy Is Involved in Vitamin E Succinate Effects on Human Gastric Carcinoma Cell Line SGC-7901 by Inhibiting mTOR Axis Phosphorylation
Source: PLoS One. 2015 Jul 13;10(7):e0132829. doi: 10.1371/journal.pone.0132829 (PMC4500415; doi:10.1371/journal.pone.0132829)

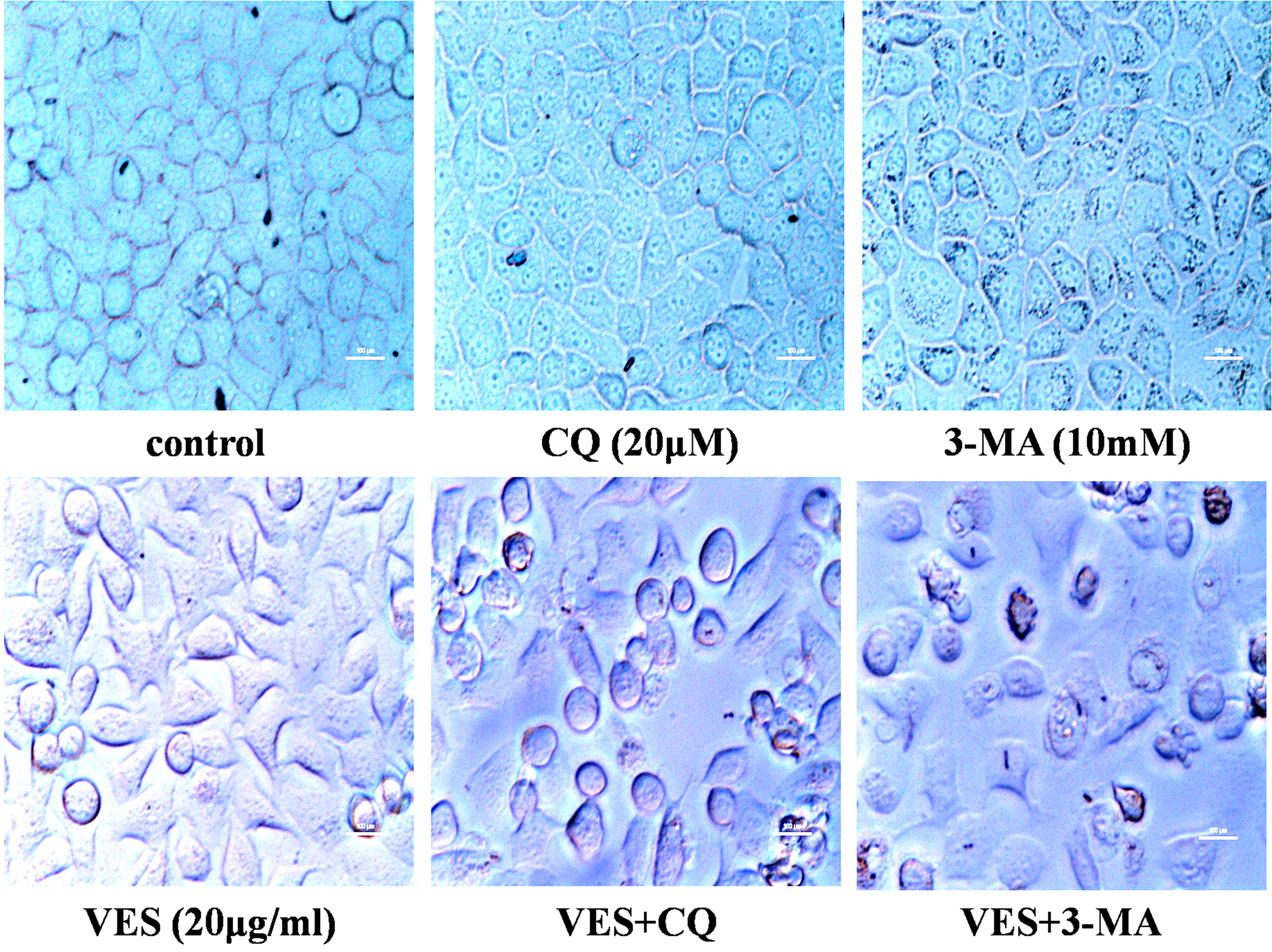

Supplement: S1 Fig — VES-treated or untreated SGC-7901 cells were exposed to 10 mM 3-MA or 20 μM CQ. The morphologies of the SGC-7901 cells were observed under a phase contrast microscope (Nikon, Japan) after 24 h of treatment. Bars: 100 μm. (TIF) [file pone.0132829.s001.tif]
